# Transcriptomic analyses of the radiation response in head and neck squamous cell carcinoma subclones with different radiation sensitivity: time-course gene expression profiles and gene association networks

**DOI:** 10.1186/s13014-016-0672-0

**Published:** 2016-07-26

**Authors:** Agata Michna, Ulrike Schötz, Martin Selmansberger, Horst Zitzelsberger, Kirsten Lauber, Kristian Unger, Julia Hess

**Affiliations:** 1 Research Unit Radiation Cytogenetics, Helmholtz Zentrum München, German Research Center for Environmental Health GmbH, 85764 Neuherberg, Germany; 2 Department of Radiotherapy and Radiation Oncology, Ludwig-Maximilians-University, 81377 Munich, Germany; 3 Clinical Cooperation Group “Personalized Radiotherapy in Head and Neck Cancer”, Helmholtz Zentrum München, 85764 Neuherberg, Germany

**Keywords:** Radioresistance, HNSCC, Head and neck cancer, Time-course gene expression, Gene association network, Signaling pathway, Differentially expressed genes, Endogenous retrovirus

## Abstract

**Background:**

Acquired and inherent radioresistance of tumor cells is related to tumor relapse and poor prognosis – not only in head and neck squamous cell carcinoma (HNSCC). The underlying molecular mechanisms are largely unknown. Therefore, systemic in-depth analyses are needed to identify key regulators of radioresistance. In the present study, subclones of the CAL-33 HNSCC cell line with different radiosensitivity were analyzed to identify signaling pathways related to the different phenotypes.

**Methods:**

Subclones with altered radiosensitivity were generated by fractionated irradiation of the parental CAL-33 cells. Differences in radiosensitivity were confirmed in colony formation assays. Selected subclones were characterized at the genomic and transcriptomic level by SKY, array CGH, and mRNA-microarray analyses. Time-course gene expression analyses upon irradiation using a natural cubic spline regression model identified temporally differentially expressed genes. Moreover, early and late responding genes were identified. Gene association networks were reconstructed using partial correlation. The Reactome pathway database was employed to conduct pathway enrichment analyses.

**Results:**

The characterization of two subclones with enhanced radiation resistance (RP) and enhanced radiosensitivity (SP) revealed distinct genomic and transcriptomic changes compared to the parental cells. Differentially expressed genes after irradiation shared by both subclones pointed to important pathways of the early and late radiation response, including senescence, apoptosis, DNA repair, Wnt, PI3K/AKT, and Rho GTPase signaling. The analysis of the most important nodes of the gene association networks revealed pathways specific to the radiation response in different phenotypes of radiosensitivity. Exemplarily, for the RP subclone the senescence-associated secretory phenotype (SASP) together with GPCR ligand binding were considered as crucial. Also, the expression of endogenous retrovirus ERV3-1in response to irradiation has been observed, and the related gene association networks have been identified.

**Conclusions:**

Our study presents comprehensive gene expression data of CAL-33 subclones with different radiation sensitivity. The resulting networks and pathways associated with the resistant phenotype are of special interest and include the SASP. The radiation-associated expression of ERV3-1 also appears highly attractive for further studies of the molecular mechanisms underlying acquired radioresistance. The identified pathways may represent key players of radioresistance, which could serve as potential targets for molecularly designed, therapeutical intervention.

**Electronic supplementary material:**

The online version of this article (doi:10.1186/s13014-016-0672-0) contains supplementary material, which is available to authorized users.

## Background

Head and neck squamous cell carcinoma (HNSCC) develops in approx. 139,000 individuals per year in Europe with a survival rate of approx. 70 % at 1 year and approx. 40 % at 5 years after therapy [1]. More than 90 % of head and neck cancers are classified as HNSCC and originate from the oral cavity, nasopharynx, oropharynx, hypopharynx, or larynx, respectively [2]. The major risk factors for HNSCC are tobacco smoking, alcohol abuse, and poor oral health [3, 4]. For oropharyngeal cancers, infection with high-risk human papilloma viruses is another important risk factor [5]. Thus, HNSCC is a very heterogeneous cancer entity also in terms of therapy response. Surgical resection followed by radio(chemo)therapy is the standard treatment of HNSCC patient [6, 7]. In locally advanced HNSCC, surgery is often limited by the complex anatomy of the affected region and, therefore, definitive radiochemotherapy is an important treatment option. However, acquired and/or inherent radioresistance of tumor cells is a common cause for tumor relapse and poor prognosis. Tumor cells derived from HNSCCs after radiotherapy have been reported to be more radioresistant than cell lines established prior to therapy, thus strengthening the clinical relevance of acquired radioresistance [8]. Along these lines, it was proposed that fractionated irradiation might preferentially eradicate radiosensitive cells, whereas radioresistant cells remain largely untouched. Accordingly, recurrent tumors mostly consist of radioresistant cells [8]. Although different potential mechanisms of radioresistance have been proposed and extensively studied, the underlying molecular details remain largely unknown [9]. Systemic in-depth analyses are needed in order to identify the master regulators of acquired radioresistance, which could serve as potential biomarkers and future therapeutic targets in novel combined modality approaches.

In this study, we characterized two subclones (#303 and #327) derived from the CAL-33 HNSCC cell line, which were generated by fractionated radiation treatment of the parental cells. CAL-33 is an HPV-negative HNSCC cell line that has been established by Gioanni et al. (1988) from a biopsy specimen prior to treatment from a squamous cell carcinoma of the tongue from a male patient [10]. The subclones derived thereof differed in radiosensitivity when compared to the parental CAL-33 cell line. Interestingly, one subclone was significantly more radioresistant, whereas the other one was significantly more radiosensitive. In order to identify potential key regulators of altered radiation sensitivity, the subclones were characterized on the genomic and transcriptomic level. Furthermore, time-course gene expression analyses were performed upon irradiation, and gene association network reconstruction and pathway enrichment analyses were utilized to identify signaling pathways related to the observed radiation phenotypes.

## Methods

### Cell culture

The human head and neck squamous cell carcinoma (HNSCC) cell line CAL-33 was obtained from the German collection of microorganisms and cell cultures (DSMZ). Cells were maintained in DMEM GlutaMAX I medium supplemented with 10 % FCS and 1 % penicillin/streptomycin and cultured at 37 °C/5 % CO_2_. Cells were mycoplasma-free as tested by the MycoAlert (Lonza) mycoplasma detection kit.

### Generation of clones with altered radiation resistance

In order to generate radioresistant subclones of the parental CAL-33 cell line, exponentially growing CAL-33 cells were exposed to fractionated irradiation (Mueller RT-250 γ-ray, tube Thoraeus Filter, 200 kV, 10 mA) according to a schedule commonly used in radiotherapy: A total dose of 20 Gy was given in daily fractions of 2 Gy 5 times per week. For each week, 2 days of recovery time were included. Afterwards, cells were cloned by limiting dilution procedure and grown for 4 to 12 weeks.

### Colony forming assay

Clonogenic survival was determined in colony formation assays as described previously [11]. Briefly, cells were seeded into 6-well plates, allowed to adhere for 4 h, and irradiated at 0, 1, 2, 4, 6 or 8 Gy, respectively (Mueller RT-250 γ-ray, Thoraeus Filter, 200 kV, 10 mA). 14 days after irradiation, colonies were fixed and stained with methylene blue. Only colonies consisting of at least 50 cells were scored. Each assay was carried out in duplicates of three different cell densities per each irradiation dose. Results from three independent experiments were subjected to linear-quadratic regression analyses employing the maximum likelihood approach. Differences between curves were evaluated using F-test [12].

### Proliferation and cell cycle analyses

Proliferation rates were determined over a period of three days upon seeding 20,000 cells per well into 24-well plates. Cells were harvested by trypsinization, and total cell numbers were determined by manual counting. Cell numbers were plotted against the growth time, and doubling times were calculated by semi-log regression analyses in the exponential growth phase. Dynamics of cell cycle distribution upon irradiation at 4 Gy was analyzed by flow cytometric phospho-histone H3(S10)/propidium iodide (PI) staining as described in [13]. Briefly, cells were collected by trypsinization and fixed in 70 % ethanol. After extensive washing, cells were stained with anti-phospho-histone-H3-Alexa488 (pH3(S10)) antibody (New England Biolabs, Frankfurt, Germany) and PI/RNase staining solution (BD Biosciences, Heidelberg, Germany). Data of 10,000 cells were recorded on an LSRII flow cytometer (BD Biosciences), and cell cycle analyses were performed by using FlowJo 7.6.5 software (Tree Star Inc., Ashland, OR, USA).

### Spectral karyotyping (SKY)

Metaphase chromosome spreads were prepared from untreated CAL-33 parental cell line and generated CAL-33 sublines. Colcemid (Roche) was added at a final concentration of 0.1 μg/ml to the culture medium of exponentially growing cells at a density of 6 × 10^6^ cells per 75 cm^2^. After 3 h of incubation time, cells were washed with PBS, trypsinized, suspended in fresh culture medium followed by hypotonic KCl treatment (75 mM) at 37 °C for 25 minutes. Following centrifugation, cells were resuspended in 2–3 ml of fixation solution and approximately 40–50 μl of cell suspension was dropped on several microscope slides. After one week of ageing at room temperature, spectral karyotyping was performed as described by Hieber et al. [14]. The karyotype of each cell line was determined based on a minimum of 15 metaphases. Chromosomal aberrations were detectable by color junctions within affected chromosomes. Spectral imaging and image analysis were performed with a SpectraCube system and SkyView imaging software (both from Applied Spectral Imaging).

### Genomic copy number typing (array CGH)

In order to characterize copy number changes of parental CAL-33 and generated CAL-33 sublines, array comparative genomic hybridization analysis (array CGH) was performed on high-resolution oligonucleotide-based SurePrint G3 Human 180 k CGH microarrays (AMADID 252206, Agilent Technologies). DNA from non-irradiated samples was isolated using the QIAamp DNA Mini Kit (Qiagen). The DNA concentration was quantified with the NanoDrop 1000 Spectrophotometer (Thermo Fisher Scientific, Germany). Slight modifications of the original Agilent array CGH protocol were introduced. 250 ng isolated cell line DNA and 250 ng sex-mismatched normal reference DNA (Promega) were labeled with Cy3 and Cy5, respectively, using the CGH labeling kit for oligo arrays (Enzo) following the Enzo’s protocol. Microcon YM-30 columns (Millipore) were used to remove the unincorporated nucleotides. Subsequent labeled DNA hybridization, washing and scanning of the CGH arrays were continued according to the Agilent’s protocol. The fluorescence intensities were extracted as text files with the Feature Extraction software 10.7 (Agilent Technologies). Obtained data were imported into the R statistical platform (version 3.2.2, www.r-project.org) and filtered for quality outliers using the QA measurements generated by the Feature Extraction software. Experimental artifacts were removed from the array CGH data using spatial normalization as suggested and described in MANOR R-package manual [15] and [16]. Array CGH profiles containing a wave bias that appear as waves in plots were removed using ridge regression based algorithms implemented in NoWaves R-package available from http://www.few.vu.nl/~mavdwiel/nowaves.html [17]. Following normalization and/or wave bias removal, data were segmented using circular binary segmentation algorithms as implemented in DNAcopy R-package [18] in order to detect breakpoints and levels in single array CGH profiles [19]. Chromosomal gains and losses were determined using CGHcall algorithm implemented in CGHcall R-package [20]. To reduce data complexity, copy number calls were transformed into regions using the R-package CGHregions [21].

### Irradiation and sample preparation

Cells were seeded into 6-well plates and allowed to adhere for 16 h. The numbers of plated cells were adjusted to the incubation times: For samples collected 0.25, 2, 7, 12, and 24 h after irradiation, 3.5 × 10^5^ cells/well were seeded, whereas for those collected after 48, 72, and 96 h, 1.75 × 10^5^ cells/well were used. Cells were irradiated at 0 or 8 Gy of gamma-irradiation (Mueller RT-250, Thoraeus Filter, 200 kV, 10 mA) at a dose rate of 1.3 Gy/min, and samples were collected after 0.25, 2, 7, 12, 24, 48, 72, and 96 h by scraping on ice. The washed, dry cell pellet was snap frozen and stored at −80 °C. Samples from 3 independent experiments were used for subsequent transcriptomic analyses. Total RNA was isolated using the Rneasy Mini Kit (Qiagen) including a DNase digestion step, according to the manufacturer’s protocol. The concentration of RNA was quantified with a Qubit 2.0 Fluorometer (Life Technologies), and RNA integrity was confirmed with a Bioanalyzer 2100 (Agilent Technologies). Samples with RNA integrity number (RIN) >7 were used in subsequent gene expression microarrays analyses.

### Global gene expression profiling

Global gene expression profiling of all CAL-33 cell lines was performed on SurePrint G3 Human Gene Expression 8x60k microarrays (Agilent Technologies, AMADID 28004) using 60 ng of total RNA according to the manufacturer’s protocol (one-color Low Input Quick Amp Labeling Kit, Agilent Technologies). Raw gene expression data were extracted as text files with the Feature Extraction software 11.0.1.1 (Agilent Technologies). All data analysis was conducted using the R statistical platform (version 3.2.2, www.r-project.org) [22]. Data quality assessment, filtering, preprocessing, normalization, batch correction based on nucleic acid labeling batches and data analyses were carried out with the Bioconductor R-packages limma, Agi4x44PreProcess and the ComBat function of the sva R-package [23–25]. All quality control, filtering, preprocessing and normalization thresholds were set to the same values as suggested in Agi4x44PreProcess R-package user guide [25]. Only HGNC annotated genes were used in the analysis. For multiple microarray probes representing the same gene the optimal probe was selected according to the Megablast score of probe sequences against the human reference sequence (http://www.ncbi.nlm.nih.gov/refseq/) [26]. If the resulted score was equal for two or more probes, the probe with the lowest differential gene expression FDR value was kept for further analyses since only one expression value per gene was allowed in subsequent gene association network (GAN) reconstruction analysis.

### Differential gene expression analysis

The time-course differential gene expression analyses were conducted between irradiated and control cells (sham-irradiated) using a natural cubic spline regression model with three degrees of freedom as described in splineTimeR R-package [27]. Obtained p-values were adjusted by the Benjamini-Hochberg method for false discovery [28]. Genes with an adjusted p-value (FDR, false discovery rate) lower than 0.05 were considered as differentially expressed and associated with radiation response. For the status quo experiment that compares the derived CAL-33 clones with the parental CAL-33 cell line, genes were considered as differentially expressed when a log2 fold-change was higher than 0.5 and a FDR value lower than 0.05.

### Identification of early and late responding genes

Temporally differentially expressed genes with fold-change above 2.0 or below 0.5 in any measured time points within the first day after irradiation were considered as early responding genes. Respectively, genes with fold-change above 2.0 or below 0.5 within the second, third or fourth day of irradiation were considered as late responding.

### Gene association network reconstruction and identification of important nodes in the reconstructed networks

Temporally differentially expressed genes were subjected to gene association network (GAN) reconstruction using a regularized dynamic partial correlation method [29]. Pairwise relationships between genes over time were inferred based on a dynamic Bayesian network model with shrinkage estimation of covariance matrices as implemented in the GeneNet R-package [30]. Analyses were conducted with a posterior probability of 0.95 for each potential undirected edge. Further, in order to determine the importance of each node in the reconstructed association networks, graph topological analyses based on centrality measures were applied [31]. Three most commonly used centrality measures: degree, shortest path betweenness and closeness describing the importance of gene in a network were combined into one centrality measure [32]. For each gene the three centrality values where ranked and the consensus centrality measure for each node was defined as the mean of the three independent centrality ranks.

### Pathway enrichment analysis

The Reactome pathway database was used to conduct the pathway enrichment analysis in order to further investigate the functions of the selected sets of differentially expressed genes [33]. Only pathways containing not more than 600 genes and not less than 20 genes were considered. Thereby, too general and too specific pathways were excluded from the analysis. Statistical significance of enriched pathways was determined by one-sided Fisher’s exact test. The resulting p-values were adjusted for FDR using the Benjamini-Hochberg method.

### qRT-PCR technical validation of gene expression data

For technical validation of the gene expression microarray data, RNA samples (500 ng) were reversely transcribed using the QuantiTect Reverse Transcription Kit (Qiagen) according to the manufacturer’s protocol and subjected to qRT-PCR reactions (10 μl) on a ViiA 7 qPCR system (Life Technologies). The following Taqman® Assays were used (Life Technologies): AKT3 (Hs00987350_m1), GADD45A (Hs01077132_m1), MAL (Hs00360838_m1), HOPX (Hs04188695_m1), HYAL3 (Hs00185910_m1), TUBGCP3 (Hs00902139_m1), RGS16 (Hs00892674_m1), TNFAIP3 (Hs00234713_m1). ACTB (Hs01060665_g1) and GAPDH (Hs99999905_m1) served as endogenous reference genes. Relative expression levels were calculated using the ΔΔCt method and Spearman correlation analyses with microarray data were performed. Validation was considered successful for Spearman’s rho > 0.5. Additionally, the fold-change values obtained from microarrays and qRT-PCR were compared.

## Results and discussion

Tumor relapse after radiochemotherapy in HNSCC is often linked to intrinsic and/or acquired radioresistance of tumor cells. However, the underlying molecular mechanisms remain largely unknown [9]. To gain knowledge on this fundamental and clinically relevant process, we established an in vitro model of acquired phenotypes of discrepant radiosensitivity in CAL-33 cells. The underlying molecular mechanisms were investigated by static and dynamic global mRNA expression analyses with subsequent network reconstruction and pathway enrichment analyses.

### CAL-33 subclones with different phenotypes of radiosensitivity and cytogenetic characteristics

**Fig. 1:**
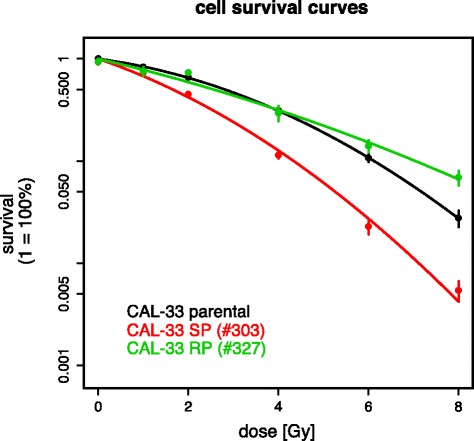
Dose-survival curves of parental CAL-33 cell line and derived subclones. The linear-quadratic cell survival curves were fitted to the measured data using maximum-likelihood method. Both subclones (SP and RP) showed statistically significant difference (*p*-values < 0.0001) in response to ionizing radiation compared to the parental CAL-33 cell line

The parental cell line CAL-33 was repeatedly irradiated in order to generate subclones with different phenotypes of radiosensitivity. To analyze acquired alterations in radiosensitivity of the derived CAL-33 subclones, long-term survival upon gamma-irradiation was assessed by colony formation assays (Fig. 1). For further analyses we selected two subclones. Both subclones #303 and #327 showed statistically significant differences (*p*-values < 0.0001) when compared to the parental CAL-33 cells. Interestingly, subclone #303 showed increased radiosensitivity (sensitive phenotype, SP), whereas subclone #327 was more radioresistant (resistant phenotype, RP) – particularly in the dose range > 4 Gy.

At first glance, the emergence of more radiation sensitive subclones appears unexpected and might be related to clonal evolution that was initiated by the irradiation-induced genomic alterations and that might occur at sublethal doses. This phenomenon has also been observed in previous studies [34, 35]. In comparison to other HNSCC cell lines, CAL-33 is very radioresistant a priori and this might explain why it appears to be very difficult to generate subclones with an even more resistant phenotype.

**Fig. 2:**
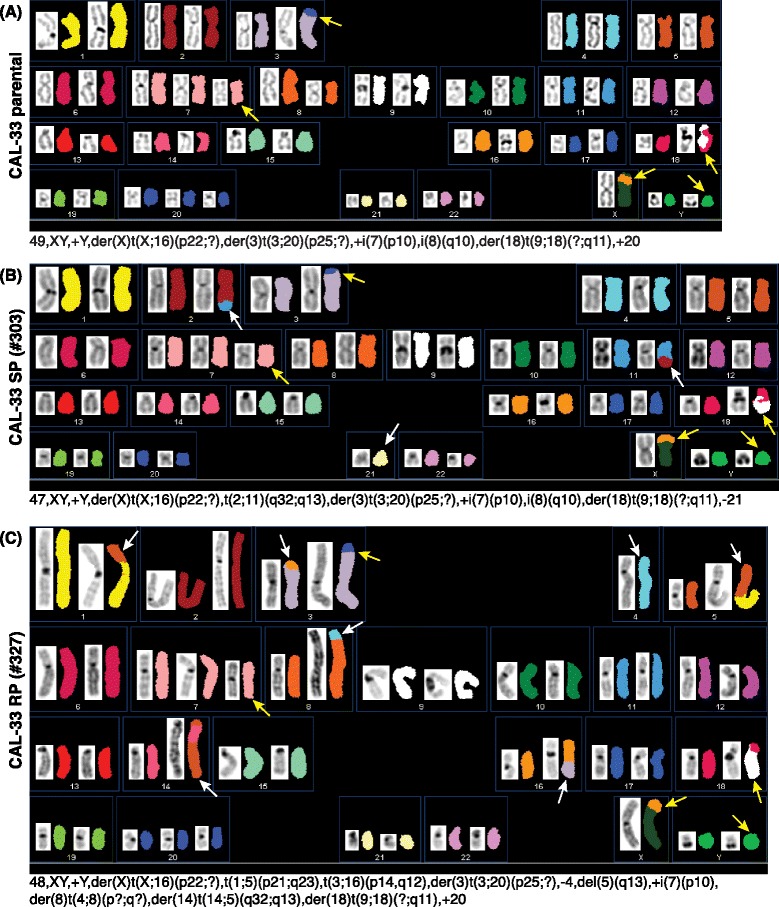
SKY results of the CAL-33 cell lines. The yellow arrows point the common for all CAL-33 cell lines marker chromosomes. The additional chromosomal rearrangements in analyzed clones compared to the parental CAL-33 cells are marked with white arrows. Structural and numerical aberrations in the parental CAL-33 cell line (**a**) involve chromosomes 3, 7, 8, 9 16, 18, 20, X, and Y. Aberrations of subclone SP (**b**) include chromosomes 2, 3, 7, 8, 9, 11, 16, 18, 20, 21, X, and Y. Chromosomal aberrations of subclone RP (**c**) affect chromosomes 1, 3, 4, 5, 7, 8, 9, 14, 16, 18, 20, X, and Y

To analyze structural and numerical chromosomal aberrations in comparison to the parental cell line, the subclones were cytogenetically characterized by SKY analyses. Structural and numerical aberrations identified by SKY in the parental CAL-33 cell line involved chromosomes 3, 7, 8, 9, 16, 18, 20, X, and Y (Fig. 2a). Structural and numerical aberrations of the SP subclone included chromosomes 2, 3, 7, 8, 9, 11, 16, 18, 20, 21, X, and Y (Fig. 2b). Chromosomes 1, 3, 4, 5, 7, 8, 9, 14, 16, 18, 20, X, and Y were affected by aberrations in subclone RP (Fig. 2c).

**Fig. 3:**
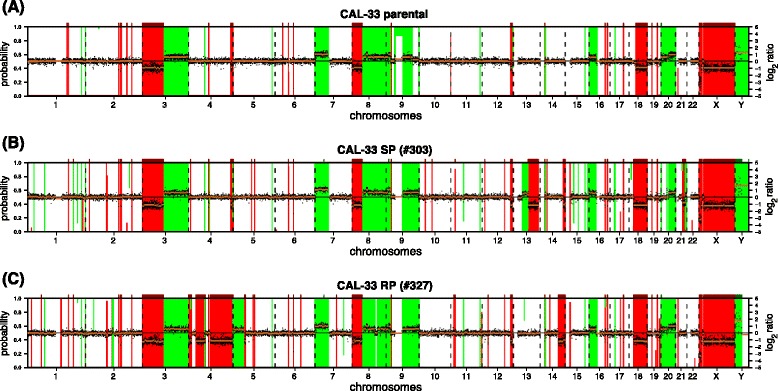
Array CGH profiles of the CAL-33 cell lines. The array CGH profiles of all of the three cell lines show copy number alterations on several chromosomes: (**a**) parental CAL-33 cell line, (**b**) subclone SP, (**c**) subclone RP. The green bars (starting from the top) represent DNA copy number gains at the corresponding position in the genome, whereas the red bars (starting from the bottom) indicate DNA copy number losses. Bars reaching beyond the middle axis (probability >0.5) were called as gains or losses

The obtained SKY results were complemented with copy number analysis by array CGH (Fig. 3 and Additional file 1: Table S1). Array CGH analysis identified 173 regions with aberrant copy number status from which 78, 111 and 132 regions were affected by DNA gains or DNA losses in the CAL-33 parental cells, SP, or RP subclones, respectively (Fig. 3 and Additional file 1: Table S1 and Additional file 2: Table S2). 68 copy number alterations (for SP) and 85 copy number alterations (for RP) were different from the parental CAL-33 cell line. Cytogenetic studies of both clones showed distinct genomic changes in comparison to the parental cell line indicating genomic key alterations for irradiation-related phenotypes on chromosomes 1, 2, 3, 4, 5, 8, 11, 14, 16 and 21. In addition, recurrent CAL-33-specific alterations on chromosomes 3, 7, 18, X and Y were observed showing the authenticity of the newly generated cell lines.

### Analysis of proliferation rates and cell cycle distribution

Proliferation rate and cell cycle distribution are important factors, which can affect radiosensitivity and/or resistance. Accordingly, we performed proliferation and cell cycle analyses. In comparison to CAL-33 parental cells, both subclones displayed prolonged doubling times (29 h for subclone SP, 30 h for subclone RP, and 24 h for the parental cell line, Additional file 3: Figure S1, A). This might be due to the observation that under exponential growth conditions, the percentage of mitotic cells in both subclones was decreased (2.1 % for subclone SP, 1.5 % for subclone RP, and 2.9 % for the parental cells), but fails to explain the differences in radiosensitivity (Additional file 3: Figure S1, B). With regard to irradiation-induced G2-arrest, the sensitive subclone SP revealed virtually identical dynamics as the parental CAL-33 cells, whereas in the resistant subclone RP G2-arrest was initially delayed, but also reached its maximum around 12 h after irradiation, and afterwards appeared to be prolonged (Additional file 3: Figure S1, C). In fact, prolonged cell cycle arrest can contribute to radioresistance as cells have more time to repair irradiation-induced DNA damage. However, extended cell cycle arrest can also be indicative for delayed DNA repair. In order to address the mechanisms underlying radioresistance on a molecular level, next we therefore performed unbiased transcriptome analyses of the CAL-33 subclones.

### mRNA gene expression analysis of the CAL-33 subclones

Microarray analyses allowed the identification of differences in basal gene expression levels between the derived subclones and the parental CAL-33 cell line. We identified 523 and 1292 differentially expressed genes (FDR < 0.05) for clones SP and RP, respectively, whereas 361 of the genes were overlapping (Additional file 4: Table S3). It is interesting to note that the RP clone exhibited more pronounced transcriptional differences than the SP clone.

Eight of the differentially expressed genes (SP and/or RP clone versus the parental CAL-33 cell line) were arbitrarily chosen for technical validation of the microarray data. Correlation analysis between qRT-PCR and microarray data showed a strong correlation for seven out of eight validated genes (Additional file 5: Table S4). The microarray and qRT-PCR derived fold-changes were in a good agreement.

Subsequently, the differentially expressed genes were subjected to pathway enrichment analyses in order to determine pathways common or specific to the radioresistant or radiosensitive phenotype. In total, 65 and 455 pathways were significantly enriched (FDR < 0.1) for the SP and RP clone, respectively (Additional file 6: Table S5). The top 100 identified pathways ordered according to the highest matching (percentage of differentially expressed genes of all genes in a pathway) were grouped into major pathways (Table 1).

**Table 1:** Significantly enriched pathways of genes differentially expressed in subclones SP and RP compared to the parental CAL-33 cells

CAL-33 SP vs parental	Common signalling pathways	CAL-33 RP vs parental
Transmembrane transport of small molecules	Signaling by VEGF	Nonhomologous end-joining (NHEJ)
GPCR ligand binding	Extracellular matrix organization	Homologous DNA pairing and strand exchange
Signaling by Rho GTPases	RNA polymerase III transcription initiation	CD28 dependent PI3K/Akt signaling
	Interferon signaling	NOTCH1 intracellular domain regulates transcription
	Signaling by interleukins	
	Senescence-associated secretory phenotype (SASP)	
	NOD1/2 signaling pathway	
	TNFR1-induced NFkB signaling pathway	
	Toll-like receptors cascades	
	Death receptor signaling	
	MAPK1/MAPK3 signaling	

Pathways common (middle) and specific to the radiosensitive (left) or radioresistant (right) phenotype are shown. The corresponding genes and their direction of regulation (up/down) are listed in Additional file 4: Table S3 and Additional file 6: Table S5

This resulted in a set of commonly deregulated pathways compared to the parental cell line but not specific for a particular phenotype of radiation sensitivity. Moreover, pathways specific to the radiosensitive or radioresistant phenotype were identified. These comprised mainly pathways that are known to be affected by ionizing irradiation in HNSCC [36–38].

### Integration of copy number changes with differentially expressed genes

To verify whether the aberrant expression of genes in the SP and RP subclones can be explained by the observed copy number changes, integration of genomic (array CGH data) and transcriptomic data was performed. For subclone SP, we identified 18 genes with DNA gains being up-regulated and 31 genes with DNA losses being down-regulated, whereas for subclone RP, 44 up-regulated genes showed copy number gains and 5 genes with copy number losses were down-regulated (Table 2).

**Table 2:** Integration of differentially expressed genes with array CGH data

CAL-33 SP (#303)			CAL-33 RP (#327)		
Gene name	FC		Gene name	FC	
PTGS1	16.417	gain	PTGS1	20.52	gain
TLR4	10.333		IL7R	10.643	
SLC2A6	7.167		SLC2A6	7.13	
TNC	5.478		SLC12A7	6.931	
PHF11	4.886		COL5A1	6.608	
PAPPA	4.594		CERCAM	6.381	
MX2	4.457		PDZD2	5.726	
LHFP	4.442		PTGER4	5.05	
RGCC	4.285		STXBP1	4.377	
GTF2F2	3.767		INPP5E	3.797	
BACE2	3.755		NKD2	3.785	
NEK3	3.564		SLC1A3	3.784	
MSANTD3	3.198		RICTOR	3.675	
FNDC3A	2.971		PTGES	3.409	
RC3H2	2.77		MVB12B	3.381	
INPP5E	2.584		CDK5RAP2	3.362	
RPL7A	2.543		PRRC2B	3.155	
UFM1	2.485		SEC16A	2.948	
SLC25A29	0.263	loss	RC3H2	2.855	
RCOR1	0.297		USP20	2.714	
HLCS	0.305		RPL7A	2.59	
CCDC85C	0.318		CARD9	2.572	
IPO5	0.322		TOR1B	2.529	
WRB	0.326		TRAF1	2.524	
PIGP	0.329		QSOX2	2.492	
CDCA4	0.36		TLR4	2.473	
ZBTB42	0.361		UGCG	2.463	
EVA1C	0.37		ZBTB43	2.396	
BTBD6	0.37		C9orf9	2.327	
TTC3	0.374		RAD1	2.255	
CLN5	0.388		TRIM32	2.241	
CCNK	0.389		PTRH1	2.221	
DSC3	0.389		CEP72	2.208	
PPP1R13B	0.389		DAP	2.035	
DYRK1A	0.393		C9orf114	2.019	
SIVA1	0.4		SDHA	2.013	
BRF1	0.404		DOLPP1	1.997	
IMPACT	0.41		RALGPS1	1.996	
IFNGR2	0.416		SURF1	1.958	
TIAM1	0.423		MTRR	1.855	
PCCA	0.426		C5orf42	1.849	
EML1	0.432		ANKRD33B	0.609	
HMGN1	0.439		OR4C6	0.576	
SETD3	0.441		NPR3	0.43	
OSBPL1A	0.462		GOLGA6L6	0.505	loss
IFNAR2	0.54		ZNF480	0.532	
LAMA3	2.1		NBPF10	1.909	
CRIP1	2.463		TPTE	2.342	
CDH2	5.373		NBPF9	2.945	

Detailed information on identified genes, their CNA status and corresponding fold changes are presented

This integrative data analysis of DNA copy number and gene expression followed by pathway enrichment analysis allowed us to identify related pathways encompassing signaling by Rho GTPases as one of the deregulated pathways in the SP subclone. At the same time we observed DNA loss and downregulation of the TIAM1 gene that belongs to the Rho GTPases signaling pathway. Yang et al. recently showed that high expression of TIAM1 is associated with poor clinical outcome in patients with HNSCC [39]. Similarly, for the RP subclone a gain and upregulation of RAD1 and RICTOR genes was observed which is in accordance with the deregulation of the homologous recombination and PI3K signaling pathways in HNSCC as previously described in [40]. For both of those clones a gain and upregulation of the TLR4 gene and the deregulation of the toll-like kinases signaling pathway including upregulation of the genes PLCG2, TAB3, RPS6KA2 has been detected. Even though contradictory reports exist, the overexpression of TLR4 and activation of related pathway has been described to promote HNSCC tumor development and to ensure tumor protection from the immune system [41].

### Time-dependent gene expression in response to ionizing radiation in CAL-33 clones

**Fig. 4:**
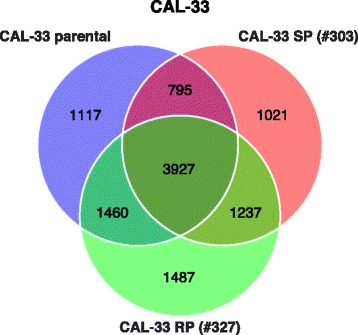
Venn diagram displaying commonly and exclusively differentially expressed genes of each CAL-33 cell line after irradiation with 8 Gy

It was hypothesized that CAL-33 subclones with different phenotypes of radiosensitivity also show differences in gene expression profiles in response to irradiation. Therefore, we performed differential time-course microarray analyses between irradiated (8 Gy) and sham irradiated control cells. 7299, 6980, and 8111 genes were differentially expressed in the CAL-33 parental, SP, and RP subclones, respectively (Table 3). Although the number of differentially expressed genes after irradiation with 8 Gy was comparable for all cell lines studied, approximately 50 % of the affected genes were not identical. More than 1000 genes were exclusively involved in the radiation response of each of the CAL-33 cell clones (Fig. 4). The entirety of differentially expressed genes in response to ionizing radiation is listed and compared for all CAL-33 cell lines in Additional file 7: Table S6.

**Table 3:** Comparison of detected and differentially expressed genes after irradiation for analyzed cell sublines

CAL-33 (8 Gy vs sham-irradiated)	Parental cell line	Subclone SP	Subclone RP
Total number of detected genes	12529	12259	12714
Number of differentially expressed genes	7299	6980	8111
Number of genes in the network	6256	5709	6859
5 % top genes	313	285	343

To identify common and separate pathways of the radiation response, pathway enrichment analyses were performed on early and late responding genes, respectively (Additional file 8: Table S7). The most interesting pathways with regard to their known role in radiation response were grouped in to major pathways (Table 4).

**Table 4:** Significantly enriched pathways of early and late responding genes after ionizing radiation

	Subclone SP	CAL-33 parental	Subclone RP
Early and late responding pathways	Apoptosis		
	Cellular senescence		
	Cell cycle		
	Cellular responses to stress		
	Signaling by Wnt		
	Signaling by Rho GTPases		
Early responding pathways	DNA double-strand break repair		
	Signaling by TGF-beta receptor complex	Interferon signaling	Cellular response to hypoxia
	TRAF6 mediated NF-kB activation		
Late responding pathways	Signaling by TGF-beta receptor complex		
	TRAF6 mediated NF-kB activation		
	Interferon signaling		
	Toll-Like receptors cascades		
	Signaling by interleukins		
	Extracellular matrix organization		
	MAPK family signaling cascades		
	NOD1/2 signaling pathway		
	PI3K/AKT signaling in cancer		
	Signaling by VEGF		
	Signaling by EGFR		
	Signaling by FGFR		
	Signaling by NOTCH		
	Signaling by ERBB2		
	Signaling by ERBB4		
	GPCR ligand binding	Base excision repair	GPCR ligand binding

It is interesting to note that all cell lines share a set of deregulated pathways (Table 4). In addition, for each of the radiosensitivity phenotypes (parental, SP, RP) specific pathways were observed. The early responses in gene expression include interferon signaling and in particular for the resistant phenotype the cellular response to hypoxia which is known for a long time to have a crucial role in radioresistance [42, 43]. Also, some other pathways from an early gene expression response that are involved in DNA repair, cell cycle, cellular response to stress and apoptosis impact on survival after irradiation and therefore contribute to radioresistance [9, 44–46].

Deregulated pathways related to late responding genes include EGFR- and PI3K/AKT signaling that were already linked to poor clinical outcome and therapy response in HNSCC [47–49] in association with ERBB2, ERBB3 and ERBB4 co-expression [50–52]. Interestingly, also involvement of ERBB2 and EERB4 receptor signaling was observed in all cell lines as a late response to ionizing radiation. Further late responding pathways include toll-like receptor cascades, interleukin signaling, NF-kB activation and interferon signaling all of which are frequently detected after treatment with ionizing radiation [53, 54]. However, the role of interleukin signaling and immune response is largely unknown so far. This also applies to cellular senescence and the impact of senescence pathways on radioresistance that were also discovered as late responding pathways in our CAL-33 subclones. Although evidence exists that senescence might be associated with the disruption of the tissue microenvironment leading to the secretion of senescence-associated pro-inflammatory factors and to the development of a pro-oncogenic environment [34] its role in radioresistance in rather unclear so far.

### Gene association network reconstruction and network analysis

To go beyond pathway enrichment analyses of differentially expressed genes, gene association networks were reconstructed. Parameters of the obtained networks (provided as igraph R-objects in Additional file 9: File S1) for all three CAL-33 cell lines are presented in Table 3. Subsequently, the combined topological centrality measure was used to characterize the biological importance of genes in the reconstructed association networks. We identified nodes (genes) that are likely to control the network by combining three network centrality measures: degree, closeness and shortest path betweenness [32, 55]. The 5 % of the highest ranked genes (listed in Additional file 10: Table S8) were mapped to the Reactome pathways to further evaluate their biological roles. The top ten pathways according to the FDR values are listed in Table 5. All identified pathways are listed in Additional file 11: Table S9. Two of the detected pathways, signaling by Rho GTPases and signaling by Wnt, were in common for all three subclones exposed to irradiation (Table 5). However, most of the pathways were different for the individual subclones. For the parental cell line, we additionally detected pathways associated with cellular response to stress, signaling by EGFR, and cytochrome P450. The most important pathways in the SP subclone were associated with axon guidance, chromatin organization and semaphorin, whereas for the RP subclone the senescence-associated secretory phenotype (SASP) together with GPCR ligand binding were considered as crucial. The co-occurrence of the SASP and GPCR signaling pathways, that are known to be connected [56], indicate that the identification of those pathways might not be accidental and suggest a high importance for the radiation resistant phenotype. In future experiments more HNSCC cell lines should be analyzed.

**Table 5:** The top pathways after mapping of 5 % highest ranked genes from the reconstructed gene association networks to the Reactome pathways

	Pathway	Genes
CAL-33 parental	Generic transcription pathway	CCNC, NR4A3, ZNF248, ZNF302, ZNF350, ZNF417, ZNF431, ZNF543, ZNF621, ZNF710, ZNF735
	Cellular responses to stress	BAG4, CDKN2D, DEDD2, GABARAPL2, HSPA1A, MAPK10, RBX1
	Diseases of signal transduction	CBL, CCNC, CTBP2, FOXO4, RBX1, TGFB1
	EPH-ephrin mediated repulsion of cells	CLTCL1, EFNA5, SRC
	Neurotransmitter release cycle	CHAT, SNAP25, STXBP1
	O-linked glycosylation	ADAMTSL5, CFP, ST3GAL3, THBS2
	Signaling by EGFR	CBL, FOXO4, NF1, PAG1, RBX1, SPRY1, SRC
	Signaling by Wnt	CTBP2, DAAM1, FRAT1, RAC2, RBBP5, RBX1, SOX3
	Signaling by Rho GTPases	ABR, CENPA, DAAM1, PKN3, RAC2, RANGAP1, SRC
	Cytochrome P450 - arranged by substrate type	CYP17A1, CYP3A4, CYP4A11
SP subclone	Axon guidance	APH1A, DPYSL3, HSP90AB1, ITGA9, LAMTOR2, NRG1, PLXNA4, PPP2CA, PSPN, RAC1, RDX, RGMB
	Generic transcription pathway	AKT2, LAMTOR2, MED26, TBL1XR1, ZNF302, ZNF394, ZNF431, ZNF561, ZNF680, ZNF691, ZNF750, ZNF774
	Cell cycle	CDKN2D, EP300, MASTL, MAU2, MCM4, NUPL2, PPP2CA, RAD21, RANGAP1, SKA2, SYCP1
	Chromatin organization	ATXN7, EP300, HIST3H2A, KDM4D, KDM5D, SUPT20H, TBL1XR1
	Diseases of signal transduction	AKT2, APH1A, BCR, CTBP2, EP300, NRG1, PPP2CA, TBL1XR1
	Signaling by Wnt	AKT2, CCDC88C, CTBP2, EP300, PLCB1, PPP2CA, RAC1, RNF146
	Semaphorin interactions	DPYSL3, HSP90AB1, PLXNA4, RAC1
	Glycolysis	ALDOC, GCK, PPP2CA
	Regulation of beta-cell development	AKT2, GCK, IAPP
	RHO GTPases activate WASPs and WAVEs	NCKIPSD, RAC1, WAS
RP subclone	Signaling by Rho GTPases	ABR, ACTB, ARHGAP35, ARHGEF7, BCR, HIST1H2BJ, HIST1H2BL, HIST2H2BE, INCENP, ITSN1, NDE1, OBSCN, RHOT1, SRGAP1, WAS
	Cell Cycle	ANAPC11, BLM, CDKN1A, CDKN2D, FZR1, HIST1H2BJ, HIST1H2BL, HIST2H2BE, INCENP, KIF23, MAU2, NDE1, NUP62, POM121, PSMC3IP, TK2, WHSC1
	Senescence-associated secretory phenotype (SASP)	ANAPC11, CDKN1A, CDKN2D, FZR1, HIST1H2BJ, HIST1H2BL, HIST2H2BE
	GPCR ligand binding	CCL19, GPR132, MC1R, MLN, OPN1SW, OPRL1, PTCH2, PTGDR, PTGER1, TAS2R14, TAS2R19, TAS2R45, TBXA2R, WNT10B
	Transcriptional regulation by small RNAs	HIST1H2BJ, HIST1H2BL, HIST2H2BE, NUP62, POM121
	G alpha (12/13) signaling events	ABR, ARHGEF7, ITSN1, OBSCN, TBXA2R
	Post-translational protein modification	ADAMTS19, ARSB, ARSG, BLM, CFP, CNIH1, CNIH2, GALNT10, NUP62, POM121, ST3GAL3
	DNA repair	ACTB, BLM, CHD1L, DTL, ERCC6, HIST1H2BJ, HIST1H2BL, HIST2H2BE, WHSC1
	Signaling by Wnt	HIST1H2BJ, HIST1H2BL, HIST2H2BE, NFATC1, PLCB1, SOX3, TCF7L2, TMED5, WNT10B
	Assembly of the primary cilium	BBS10, CC2D2A, NDE1, NPHP1, PDE6D, TCTEX1D2, TTC30B

### Expression of endogenous retrovirus and related pathways

**Fig. 5:**
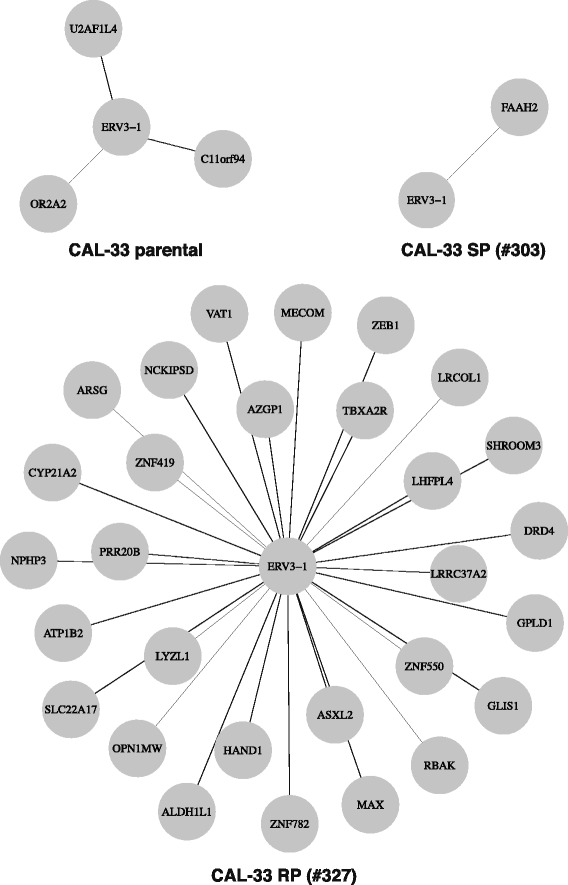
First neighborhood of the ERV3-1 gene extracted from the reconstructed gene association networks

The analysis of differentially expressed genes between the RP and SP subclones and the parental cell line revealed the ERVMER34-1 gene as differentially expressed in both clones. Approximately 8 % of the human genome consists of endogenous retroviruses (ERVs) that have been derived from exogenous retroviruses following infection and DNA integration into germ line cells [57, 58]. Although most of the ERV sequences have defective structures, some of ERV genes still have an open reading frame (ORF) and protein expression [59]. ERV genes can promote homologous and non-homologous recombination and therefore, may introduce new mutations [60, 61]. Furthermore, ERVs may lead to genome instability, and contribute to tumor initiation and progression [62]. The expression of ERV genes has been demonstrated in various cancers, including breast, ovarian, prostate and melanoma [63–66]. The differential gene expression analyses of the subclones in comparison to the parental CAL-33 cells revealed ERVMER34-1 gene as differentially expressed in both derived clones (Additional file 4: Table S3). Subsequently, we tested whether any of the retroviral genes were differentially expressed in response to ionizing radiation. Apart from ERVMER34-1, we were able to identify another endogenous retroviral gene, ERV3-1, that was temporally differentially expressed following radiation. The time dependent expression of those genes suggests ERV3-1 expression to be associated with the radiation response, whereas ERVMER34-1 expression exhibits rather random fluctuations (Additional file 12: Figure S2 and Additional file 13: Figure S3). Thus, the ERV3-1 expression clearly implies a possible association with the radiation exposure. The radiation-associated upregulation of ERV3-1 is demonstrated for all subclones starting from the second day of irradiation. To our knowledge, the expression of ERV3-1 following radiation and its influence on radiation resistance has not been addressed in detail so far. A recent study by Lee et al. [67] has demonstrated an increase in the expression of ERV3-1 (HERV-R) *env* related to a fractionated exposure to γ-radiation in radioresistant A549 lung cancer cells but not in less radioresistant H460 cells. The presented results raise the question whether overexpression of ERV3-1 might be involved in the radiation response of HNSCC cells. To gain knowledge about the potential gene interactions with the ERV3-1 gene we used the gene association networks reconstructed for all CAL-33 subclones and extracted the putative direct or indirect ERV3-1 interaction partners resulting in the first neighborhood genes of ERV3-1 differ between the three analyzed cell lines (Fig. 5). The largest first neighborhood gene association network can be observed for the RP subclone where 29 genes are linked to ERV3-1. For the CAL-33 parental cell line and the SP subclone the first neighborhood gene association network consist only of three (OR2A2, U2AF1L4, C11orf94) and one (FEEH2) potential association partners, respectively. The considerably larger first neighborhood of the ERV3-1 gene for the RP cells suggests a more important role of this gene for acquired radiation resistance. In addition, a Reactome pathway enrichment analysis revealed that the first neighborhood genes of the ERV3-1 gene in RP cells were associated with GPCR signaling (DRD4, OPN1MW, TBXA2R), transmembrane transport of small molecules (ATP1B2, AZGP1, SLC22A17), generic transcription pathway (ZNF419, ZNF550, ZNF782), signaling by Rho GTPases (NCKIPSD), and cell cycle (MAX). However, to our knowledge, the interaction partners of the ERV3-1 gene have not been studied in detail so far, which makes an interpretation difficult and highly speculative at this time. Also further studies have to be performed in order to validate the gene associations with ERV3-1independently.

## Conclusion

In conclusion, the present study presents comprehensive gene expression data of CAL-33 subclones of different radiosensitivity. Based on these data networks have been identified that are linked to the radiation response phenotypes. The pathways associated with the resistant phenotype are of special interest focusing on the senescence-associated secretory phenotype (SASP) together and GPCR ligand binding. Also, the radiation-associated expression of the endogenous retrovirus ERV3-1 appears highly attractive for further studies on the molecular mechanisms of acquired radioresistance.

## Abbreviations

CGH, comparative genomic hybridization; CNA, copy number aberration; ERV endogenous retrovirus; FDR, false discovery rate; GAN, gene association network; HNSCC, head and neck squamous cell carcinoma; RP, resistant phenotype; SKY, spectral karyotyping; SP, sensitive phenotype

## Additional files

Additional file 1: Table S1. Regions of copy number changes identified by array CGH analysis. Array CGH analysis identified 173 regions with copy number changed. 78, 111 and 132 regions were identified as gains or losses for the parental cell line, the SP and RP subclones, respectively. Regions with normal copy number status are labeled with “0”, DNA-gains are marked with “1” and DNA-losses with “-1”. Regions with different copy number variations between clones SP and RP and the parental cell line are marked as “diff”. (XLSX 56 kb) Additional file 2: Table S2. Overview on the cytogenetic aberrations in CAL-33 cells. Clonal structural and numerical chromosomal aberrations detected by SKY and copy number changes detected by array CGH are listed. (DOCX 94 kb) Additional file 3: Figure S1. Analyses of proliferation rates and cell cycle distribution. Proliferation curves of the CAL-33 parental cell line and the subclones SP and RP are shown in panel (A). Doubling times were determined in the exponential growth phase by semi-log regression. Cell cycle analyses were performed under exponential growth conditions (B). The dynamics of G2-arrest upon irradiation at 4 Gy are shown in figure panel (C). (PDF 318 kb) Additional file 4: Table S3. Differentially expressed genes and the corresponding fold-changes. (XLSX 117 kb) Additional file 5: Table S4. Technical validation of microarray data. Eight of the differentially expressed genes detected with the microarray technology were arbitrarily chosen for technical validation by qRT-PCR. Correlation analysis results (Spearman’s rho coefficient) and fold-changes (microarray and qRT-PCR) are shown. Asterisks (*) indicate fold-change values for genes detected as differentially expressed by microarray analysis. (DOCX 52 kb) Additional file 6: Table S5. Pathway enrichment analysis results of differentially expressed genes in clones SP and RP in comparison to parental CAL-33 cells. (XLSX 46 kb) Additional file 7: Table S6. Differentially expressed genes in response to irradiation with 8 Gy for all CAL-33 cell lines. Differentially expressed genes are listed together with the corresponding fold-changes. Additionally, commonly and exclusively differentially expressed genes have been included. (XLSX 2575 kb) Additional file 8: Table S7. Significantly enriched pathways of early and late responding genes after irradiation with 8 Gy. Pathways with FDR < 0.1 were considered statistically significant. (XLSX 102 kb) Additional file 9: File S1. Reconstructed gene association networks. All obtained gene association networks are provided as R-objects of type igraph. (RDATA 32863 kb) Additional file 10: Table S8. 5 % most important genes identified by centrality measures. The 5 % highest ranked genes from the reconstructed gene association networks together with corresponding ranks of centrality measures are shown. (XLSX 88 kb) Additional file 11: Table S9. Involved pathways after mapping of 5 % highest ranked genes from the gene association networks to the Reactome pathways. The pathways together with the mapped most important (5 % highest ranked) genes are inluded. (XLSX 92 kb) Additional file 12: Figure S2. Temporal expression and corresponding fold-changes of gene ERVMER34-1 after irradiation with 8 Gy. (PDF 535 kb) Additional file 13: Figure S3. Temporal expression and corresponding fold-changes of gene ERV3-1 after irradiation with 8 Gy. (PDF 531 kb)
