# Supplementary material for: Transcriptomic analyses of the radiation response in head and neck squamous cell carcinoma subclones with different radiation sensitivity: time-course gene expression profiles and gene association networks
Source: Radiat Oncol. 2016 Jul 26;11:94. doi: 10.1186/s13014-016-0672-0 (PMC4960706; doi:10.1186/s13014-016-0672-0)
Supplement: Additional file 2: Table S2. — Overview on the cytogenetic aberrations in CAL-33 cells. Clonal structural and numerical chromosomal aberrations detected by SKY and copy number changes detected by array CGH are listed. (DOCX 94 kb) [file 13014_2016_672_MOESM2_ESM.docx]

| **cell line** | **clonal structural and numerical chromosomal aberrations detected by SKY** | **clonal copy number changes detected by aCGH** | |
| --- | --- | --- | --- |
|  |  | **DNA gains** | **DNA losses** |
| CAL-33 parental | 49, XY, +Y, der(X)t(X;16)(p22;?), der(3)t(3;20)(p25;?), +i(7)(p10), i(8)(q10), der(9;18)(?;q11), +20 | 1q42.2, 1q44, 3q11.1-q29, 4q13.2, 5q34, 7p22.3-p11.2, 8q11.1-q24.3, 9p24.3-p22.2, 9p13.3-p13.1, 9q21.11-q22.33, 9q31.1-q31.3, 9q34.3, 11q24.2, 11q24.33, 14q11.2, 15q25.2, 16p13.3-p11.2, 16p11.1, 17p11.2, 20p13-p11.23, 20p11.22-p11.1, 20q11.21-q13.33, 20p13 | 1q24.2, 1q25.1, 2q22.1, 2q23.1, 2q23.3, 2q31.1, 2q33.1, 2q37.3, 3p26.3-p11.1, 3q26.1, 4q21.3, 4q34.3-q35.2, 6p21.32, 6p12.1, 6q14.1, 8p23.3-p11.1, 9p21.3, 10q26.3, 12q24.31-q24.33, 14q11.2, 16q22.1, 16q23.1, 17q23.1, 18q12.1, 18q12.2-q12.3, 18q12.3-q23, 19p12, 19q13.31 |
| CAL-33 SP (#303) | 47, XY, +Y, der(X)t(X;16)(p22;?), t(2;11)(q32;q13), der(3)t(3;20)(p25;?), +i(7)(p10), i(8)(q10), der(9;18)(?;q11), -21 | 1p36.11, 1p31.1, 1q31.3, 1q32.3-q41, 1q42.2, 1q44, 3q11.1-q29, 4q13.2, 4q22.1, 4q35.2, 5q34, 7p22.3-p11.2, 8q11.1-q24.3, 9p24.3-p22.2, 9q21.11-q34.3, 11q11, 11q24.2, 13q13.3-q21.31, 14q11.2, 15q14, 15q21.3, 15q25.2, 15q26.2, 16p13.3-p11.2, 17p11.2, 20p13, 20p11.21-p11.1, 20q11.22-q13.33, 21q22.2-q22.3 | 1q25.1, 1q31.3, 2p11.2-p11.1, 2q22.1, 2q23.1, 2q23.3, 2q33.1, 2q37.3, 2q33.1, 3p26.3-p11.1, 4p16.1, 4q21.3, 4q34.3-q35.2, 5p15.33, 5q14.1, 5q15, 6p12.1, 6p11.2, 6q14.1, 8p23.3-p11.1, 9p21.3, 10q21.1, 11p15.1, 12p13.2, 12q23.1, 12q24.31-q24.33, 13q21.31-q33.3, 14q11.2, 14q12, 14q32.2-q32.33, 15q11.2, 16q22.1, 16q23.1, 17q23.1, 18q11.2-q23, 19p12, 19q13.31, 21q21.3-q22.2, |
| CAL-33 RP (#327) | 48, XY, +Y, der(X)t(X;16)(p22;?), t(1;5)(p21;q23), t(3;16)(p14,q12), der(3)t(3;20)(p25;?), -4, del(5)(q13), +i(7)(p10), der(8)t(4;8)(p?;q?), der(14)t(14;5)(q32;q13), der(9;18)(?;q11), +20 | 1p31.1, 1q42.2, 1q44, 2p22.3, 2q13-q14.1, 3q11.1-q29, 4q13.2, 5p15.33-p11, 5q34, 7p22.3-p11.2, 8q11.1-q22.1, 8q23.1-q24.12, 8q24.13-q24.3, 9p24.3-p22.2, 9q21.11-q34.3, 11q11, 11q24.2, 14q11.2, 15q25.2, 16p13.3-p11.1, 17p11.2, 20p13-p11.1, 20q11.21-q13.33 | 1p36.13, 1p32.2, 1q21.1, 1q25.1, 1q31.3, 1q44, 2p11.2-p11.1, 2q13, 2q22.1, 2q23.1, 2q23.3, 2q33.1, 2q37.3, 3p26.3-p11.1, 4p16.3-p15.33, 4p15.1-q13.3, 4q21.3, 4q22.2-q35.2, 5q11.1-q11.2, 5q14.3, 5q15, 6p12.1, 6p11.2, 6q14.1, 6q21, 7q21.2, 8p23.3-p11.1, 9p21.3, 11p15.2-p15.1, 11q25, 12p12.1, 12q23.1, 12q24.31-q24.33, 14q11.2, 14q21.1, 14q24.3-q32.33, 15q11.1-q11.2, 16q22.1, 16q23.1, 17q21.31, 17q23.1, 18q12.1-q23, 19p12, 19q13.31, 19q13.41-q13.42, 21p11.2-p11.1 |
