# Supplementary figures and images for: Transcriptomic analyses of the radiation response in head and neck squamous cell carcinoma subclones with different radiation sensitivity: time-course gene expression profiles and gene association networks
Source: Radiat Oncol. 2016 Jul 26;11:94. doi: 10.1186/s13014-016-0672-0 (PMC4960706; doi:10.1186/s13014-016-0672-0)

(A)

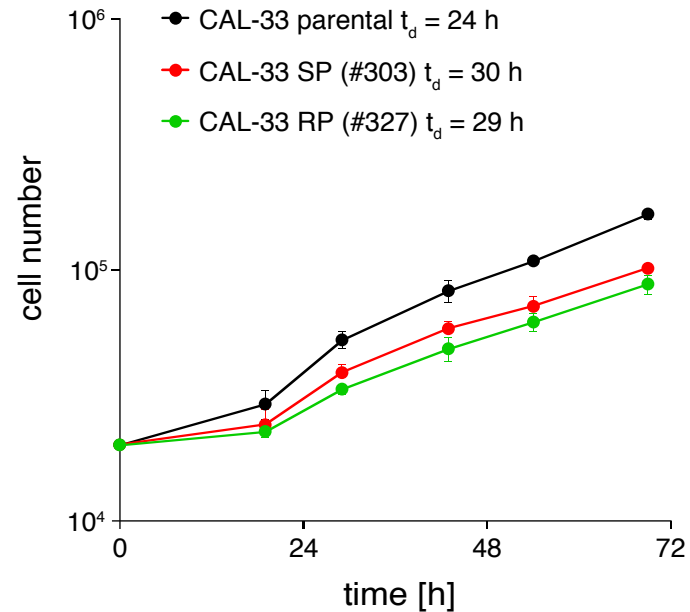

(B)

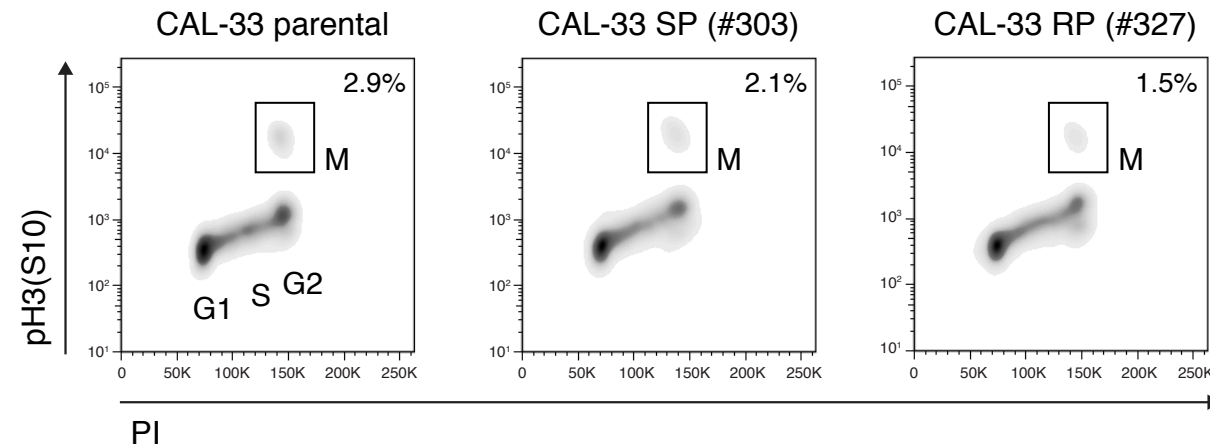

(C)

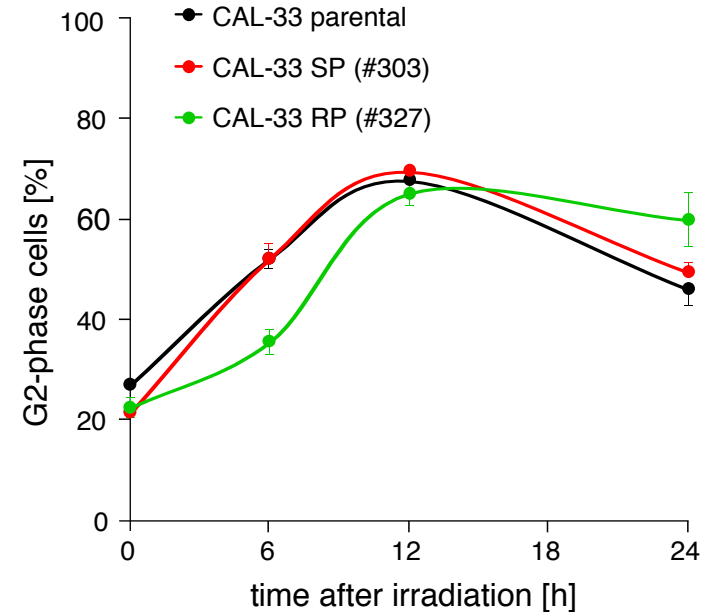

Supplement: Additional file 3: Figure S1. — Analyses of proliferation rates and cell cycle distribution. Proliferation curves of the CAL-33 parental cell line and the subclones SP and RP are shown in panel (A). Doubling times were determined in the exponential growth phase by semi-log regression. Cell cycle analyses were performed under exponential growth conditions (B). The dynamics of G2-arrest upon irradiation at 4 Gy are shown in figure panel (C). (PDF 318 kb) [file 13014_2016_672_MOESM3_ESM.pdf]

CAL-33 parental

ERVMER34-1

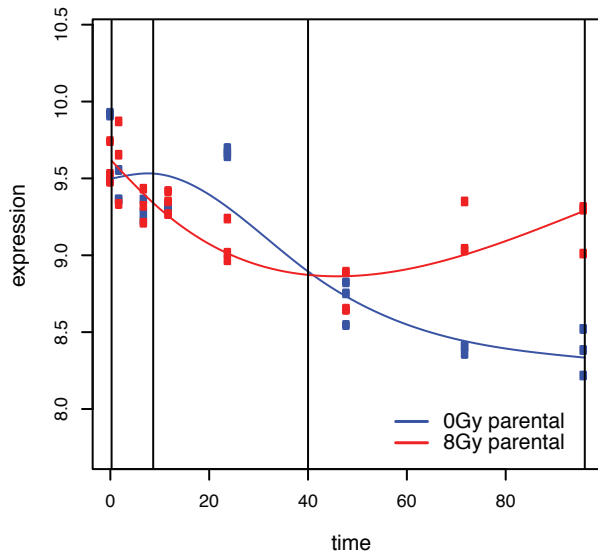

ERVMER34-1

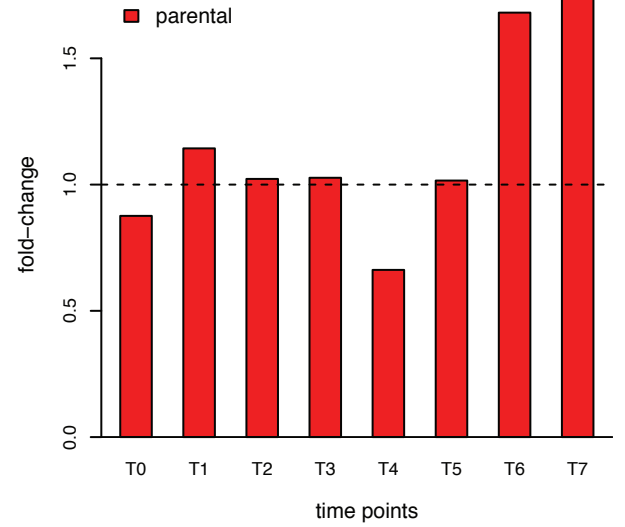

CAL-33 SP (#303)

ERVMER34-1

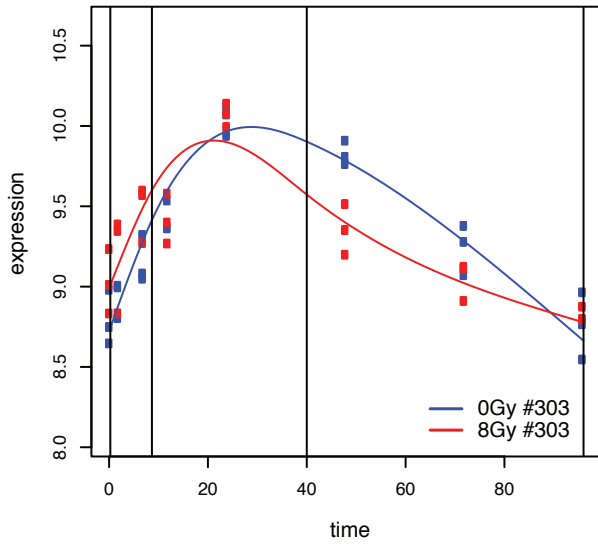

ERVMER34-1

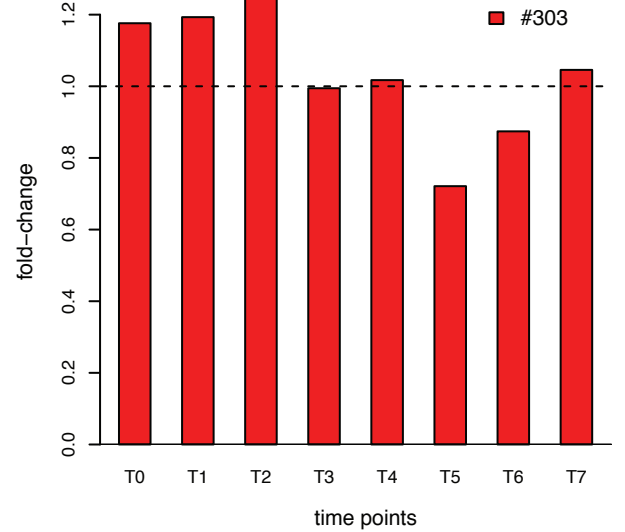

CAL-33 RP (#327)

ERVMER34-1

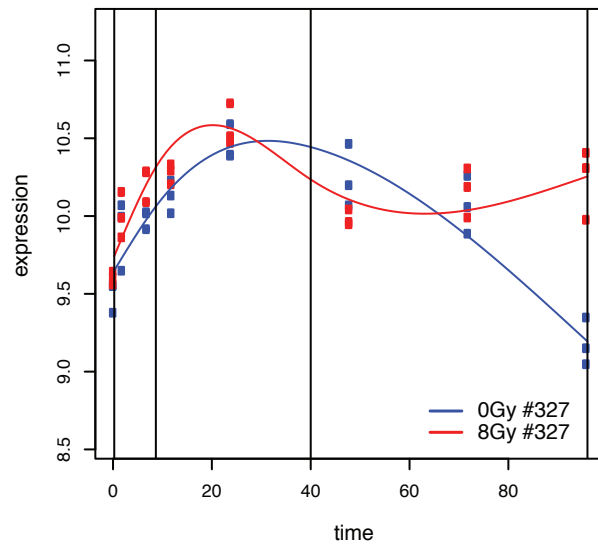

ERVMER34-1

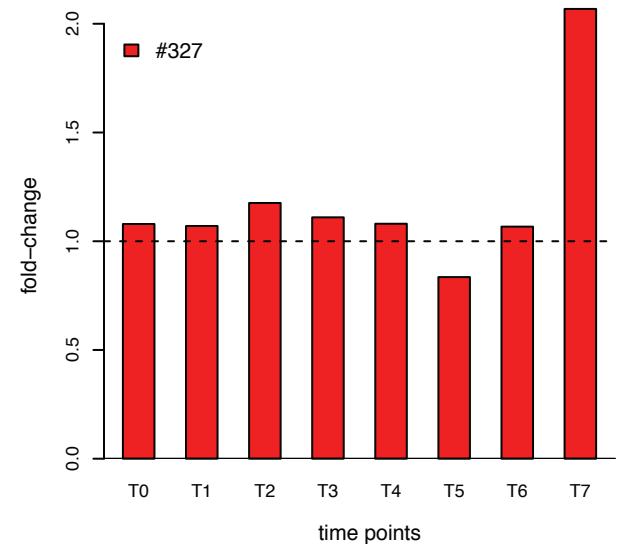

Supplement: Additional file 12: Figure S2. — Temporal expression and corresponding fold-changes of gene ERVMER34-1 after irradiation with 8 Gy. (PDF 535 kb) [file 13014_2016_672_MOESM12_ESM.pdf]

CAL-33 parental

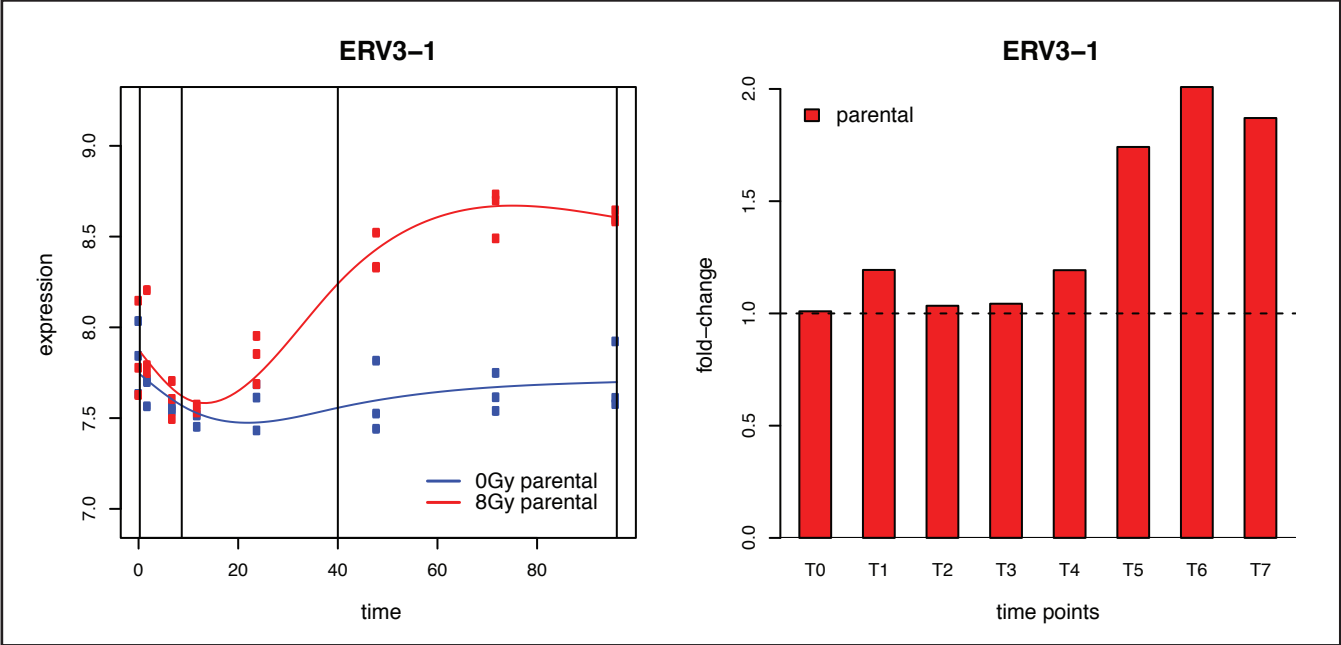

CAL-33 SP (#303)

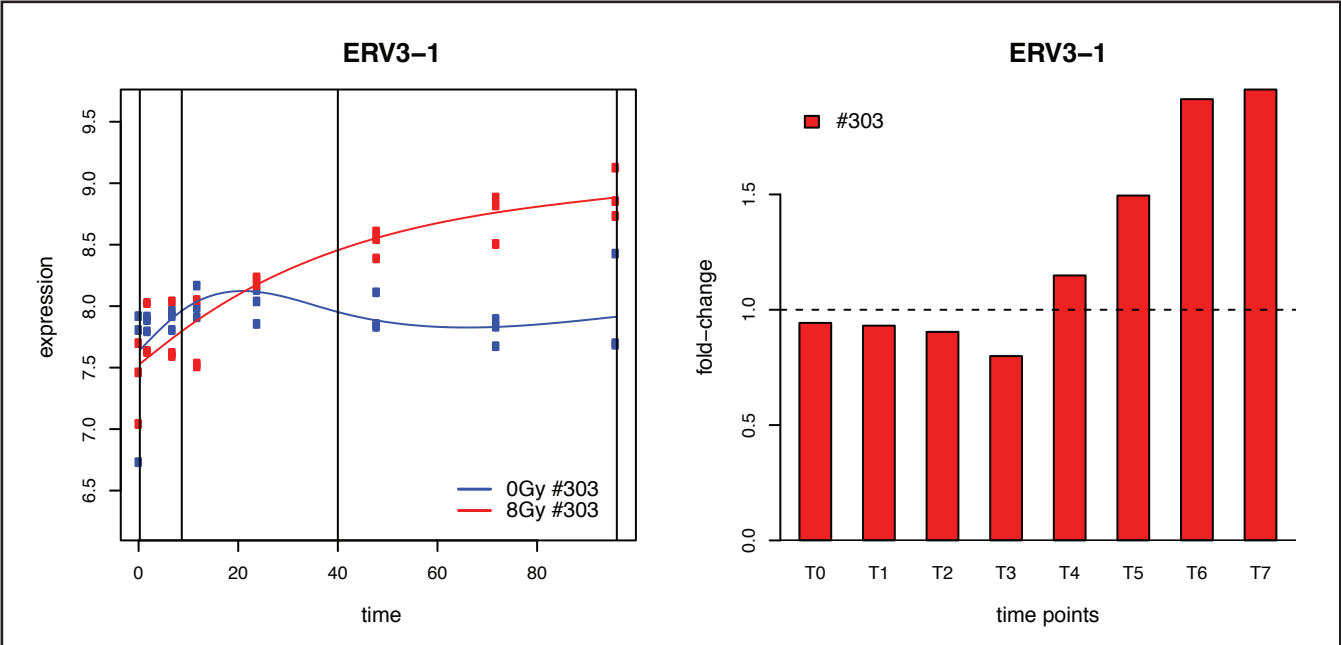

CAL-33 RP (#327)

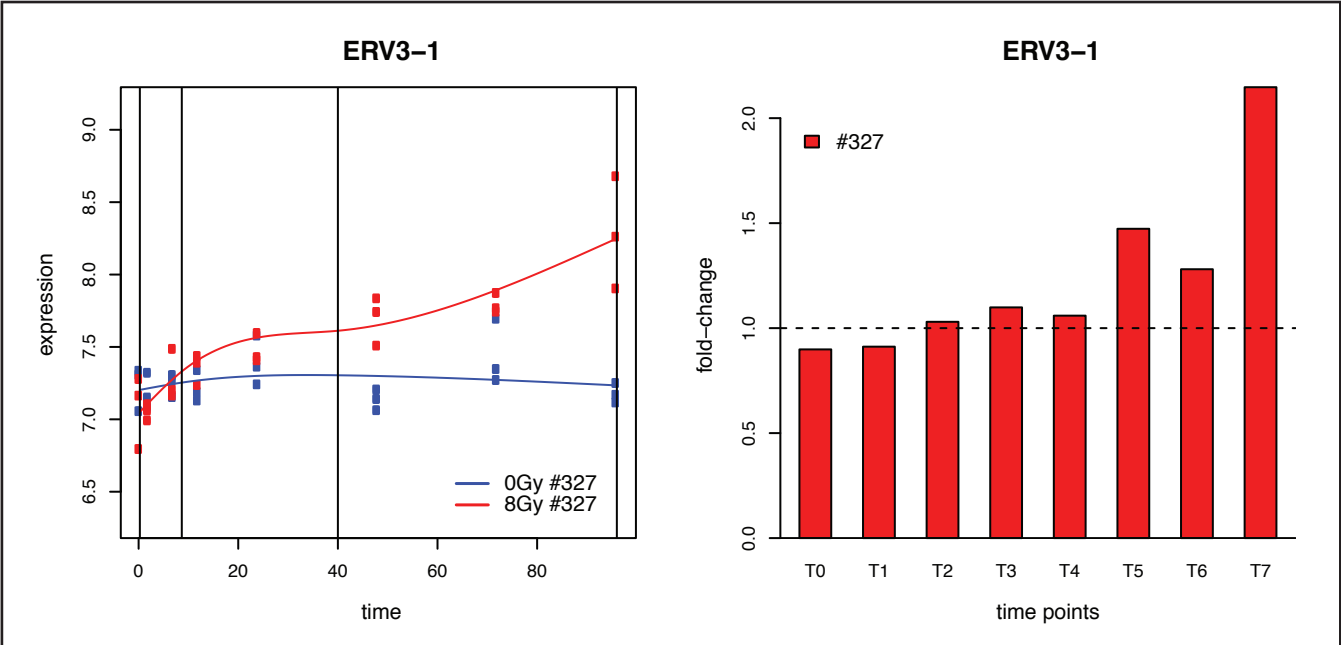

Supplement: Additional file 13: Figure S3. — Temporal expression and corresponding fold-changes of gene ERV3-1 after irradiation with 8 Gy. (PDF 531 kb) [file 13014_2016_672_MOESM13_ESM.pdf]
