# Supplementary material for: Transcriptomic analyses of the radiation response in head and neck squamous cell carcinoma subclones with different radiation sensitivity: time-course gene expression profiles and gene association networks
Source: Radiat Oncol. 2016 Jul 26;11:94. doi: 10.1186/s13014-016-0672-0 (PMC4960706; doi:10.1186/s13014-016-0672-0)
Supplement: Additional file 5: Table S4. — Technical validation of microarray data. Eight of the differentially expressed genes detected with the microarray technology were arbitrarily chosen for technical validation by qRT-PCR. Correlation analysis results (Spearman’s rho coefficient) and fold-changes (microarray and qRT-PCR) are shown. Asterisks (*) indicate fold-change values for genes detected as differentially expressed by microarray analysis. (DOCX 52 kb) [file 13014_2016_672_MOESM5_ESM.docx]

| **gene** | **Spearman’s rho correlation coefficient** | **fold-change SP (#303) vs parental** | | **fold-change RP (#327) vs parental** | |
| --- | --- | --- | --- | --- | --- |
|  |  | **qRT-PCR** | **microarray** | **qRT-PCR** | **microarray** |
| AKT3 | 0.54 | 5.20 | 2.84* | 4.22 | 1.73 |
| GADD45A | 0.94 | 3.10 | 3.02* | 1.73 | 1.82 |
| MAL | 1 | 5.34 | 2.48* | 2.98 | 1.69 |
| HOPX | 0.77 | 0.95 | 1.28 | 0.11 | 0.22* |
| HYAL3 | 0.89 | 1.15 | 1.20 | 3.44 | 2.02* |
| TUBGCP3 | 0.43 | 0.78 | 0.69 | 0.85 | 0.53* |
| RGS16 | 0.83 | 0.26 | 0.19* | 0.72 | 0.43* |
| TNFAIP3 | 0.89 | 3.43 | 4.74* | 6.41 | 6.29* |

*Significantly differentially expressed genes (FDR < 0.05) detected with the microarray technology
